# Effects of flaxseed oil on anti-oxidative system and membrane deformation of human peripheral blood erythrocytes in high glucose level

**DOI:** 10.1186/1476-511X-11-88

**Published:** 2012-07-08

**Authors:** Wei Yang, Juan Fu, Miao Yu, Qingde Huang, Di Wang, Jiqu Xu, Qianchun Deng, Ping Yao, Fenghong Huang, Liegang Liu

**Affiliations:** 1 Department of Nutrition and Food Hygiene, School of Public Health, Tongji Medical College, Huazhong University of Science and Technology, 13 Hangkong Road, Wuhan, 430030, China; 2 Hubei Key Laboratory of Food Nutrition and Safety, School of Public Health, Tongji Medical College, Huazhong University of Science and Technology, 13 Hangkong Road, Wuhan, 430030, China; 3 Oil Crops Research Institute, Chinese Academy of Agricultural Science, 2-Xudong Road, Wuhan, 430062, China

**Keywords:** Flaxseed oil, Antioxidation, Erythrocytes, Diabetes, Membrane

## Abstract

**Background:**

The erythrocyte membrane lesion is a serious diabetic complication. A number of studies suggested that n-3 fatty acid could reduce lipid peroxidation and elevate α- or γ-tocopherol contents in membrane of erythrocytes. However, evidence regarding the protective effects of flaxseed oil, a natural product rich in n-3 fatty acid, on lipid peroxidation, antioxidative capacity and membrane deformation of erythrocytes exposed to high glucose is limited.

**Methods:**

Human peripheral blood erythrocytes were isolated and treated with 50 mM glucose to mimic hyperglycemia in the absence or presence of three different doses of flaxseed oil (50, 100 or 200 μM) in the culture medium for 24 h. The malondialdehyde (MDA) and L-glutathione (GSH) were measured by HPLC and LC/MS respectively. The phospholipids symmetry and membrane fatty acid composition of human erythrocytes were detected by flow cytometry and gas chromatograph (GC). The morphology of human erythrocyte was illuminated by ultra scanning electron microscopy.

**Results:**

Flaxseed oil attenuated hyperglycemia-induced increase of MDA and decrease of GSH in human erythrocytes. Human erythrocytes treated with flaxseed oil contained higher C22:5 and C22:6 than those in the 50 mM glucose control group, indicating that flaxseed oil could reduce lipid asymmetric distribution and membrane perturbation. The ultra scanning electron microscopy and flow cytometer have also indicated that flaxseed oil could protect the membrane of human erythrocytes from deformation at high glucose level.

**Conclusion:**

The flaxseed oil supplementation may prevent lipid peroxidation and membrane dysfunction of human erythrocytes in hyperglycemia.

## Background

The α-linolenic acid (ALA), the n-3 polyunsaturated fatty acid (PUFA) existed in vegetable oils such as flaxseed oil, is an essential fatty acid for humans [1]. In the human body, ALA can be converted into longer-chain n-3 PUFA including eicosapentaenoic acid (EPA) and docosahexaenoic acid (DHA) [2]. Previous studies indicated that dietary n-3 PUFA may possess protective effects against cardiovascular disease (CVD) [3]. The Greenland Eskimos and Japanese have a high dietary intake of long-chain n-3 PUFA from seafood and a low incidence of CVD [3]. An earlier study found that n-3 PUFA diets reduced the sequelae of cerebral and myocardial infarction in experimental animals [4]. In addition, n-3 PUFA supplementation could increase the α- and γ-tocopherol contents of the red blood cell membranes and membrane fluidity [5].

Oxidative stress is considered as a common cause of diabetes mellitus (DM) [6]. A number of studies found that severe malformation and high lipid peroxidation in erythrocytes are evident markers in diabetic animals and patients [7,8]. At high blood glucose level, erythrocytes could easily deform into acanthoid cells (acanthocytes) which is difficult to flow through the blood vessels or microvessels [7,8]. This kind of cellular deformation was probably related to hyperglycemia, protein glycation, sorbitol accumulation or dyslipidemia [8]. The hyperglycemia or dyslipidemia easily induce serious oxidative stress that causes serious cellular dysfunction as well as hematic and vascular complications in diabetic patients [6-9]. Previous researches have illustrated that PUFA could reduce cellular dysfunction or other complications of DM. For instance, ALA supplements for 12 weeks elevated EPA and DHA contents of erythrocyte in healthy people [10,11]. Moreover, it has been observed in clinical research that ALA remarkably reduced lipid peroxidation in serum of type-1 diabetic patients [12]. Zeman et al reported that the concentrations of conjugated dienes in low-density lipoprotein (LDL) increased significantly and the levels of triglycerides, total homocysteine and microalbuminuria decreased significantly after diabetic patients ingested n-3 PUFA (Omega-3 Forte, 3.6 g/day) and received statin-fibrate treatment (Pravastatin 20 mg + micronized Fenofibrate 200 mg/day) [13]. Nettleton et al reported that fish oil supplementation (4 g/day) and corn oil capsules supplementation (1 g oil and 13.4 mg α-tocopherol in each capsules) both significantly increased levels of LDL- malondialdehyde (MDA) adduct in type-2 diabetic patients [14]. However, studies regarding the effect of flaxseed oil on antioxidation and protecting membrane of erythrocytes at high glucose level are limited.

Therefore, this study aims to use human peripheral blood erythrocytes to test the hypothesis that flaxseed oil supplement can reduce oxidative stress and protect membrane of erythrocytes exposed to high glucose level in vitro.

## Materials and methods

### Materials

Thiobarbituric-acid (TBA), L-glutathione reduced (GSH, 99%), L-glutathione oxidized (GSSG, 98%), trifluoroacetic acid, borontrifluoride (BF_3_) / methanol (MeOH) and hexane were purchased from Sigma-Aldrich (St. Louis, MO, U.S.A). Annexin V-FITC kit was brought from Keygen biotech company (Nanjing, China).

#### Blood sample collection and preparation

Blood samples were collected from 96 healthy and non-smoking volunteers (48 males aged 28.21 ± 3.11 years old and 48 females aged 27.76 ± 2.94 years old). Peripheral blood samples were collected into tubes containing ethylenediamine tetraacetic acid (EDTA). The volunteers were recruited from the clinical department of Hubei Provincial Center for Disease Control and Prevention (Wuhan, China). Informed consent was obtained from each individual. All procedures were approved by the Medical Ethics Committee of Tongji Medical College. The EDTA-blood mixture was centrifuged at 350 × g for 10 min (5804R, Eppendorf AG, Hamburg, Germany). The clear plasma and buffy coat layers were carefully discarded. The erythrocyte suspension was washed with cold sodium chloride solution (0.15 M).

### Flaxseed oil preparation, human erythrocytes culture and study design

Flaxseed oil was kindly provided by Oil Crops Research Institute-Chinese Academy of Agricultural Science (The fatty acid compositions of flaxseed oil demonstrated in Additional file 1: Table S1). Preparations of stock solution of flaxseed oil and erythrocytes culture were carried out following the protocol described in earlier studies [14,15]. Briefly, flaxseed oil was dissolved in ethanol to three concentrations (50, 100 and 200 μM) and Phosphate Buffered Saline (PBS) was used as culture media (0.01 M, pH = 7.4). In certain experiments, the erythrocyte suspension (400 μL) was pre-incubated with three concentrations of flaxseed oil for 4 h, and then the glucose stock solution (50 mM) was added to cell suspension and incubated for 20 h. The PBS and 50 mM glucose without flaxseed oil served as negative control group and positive control group respectively. The contents of the flasks were incubated in a shaking water bath at 37°C. Percentage of hemolysis was less than 1% in all incubations. The erythrocytes were washed twice with a 1-to-10 dilution with NaCl (0.15 M) before biochemical analysis. All incubations contained 10 μL penicillin-streptomycin/ml cell suspension to vitiate any microbial growth during the overnight incubations. The working solution of penicillin-streptomycin contained 100 U/mL penicillin G and 100 μg/mL streptomycin in buffer.

### MDA assayed by high performance liquid chromatography (HPLC)

MDA, a widely used indicator of lipid peroxidation, was assessed by HPLC, based on a previous report with some modifications [6,15]. For this purpose, erythrocytes (0.4 mL) were suspended in PBS (0.8 mL). The butylated hydroxytoluene (0.025 mL, 88 mg BHT/10 mL absolute alcohol) and trichloroacetic acid (TBA, 0.5 mL, 30%) were also added in cell suspension. Then, the tubes were vibrated on vortex and incubated on ice for 2 h. After incubation, the tubes were centrifuged at 334 × g for 15 min (5804R, Eppendorf AG, Hamburg, Germany). For each sample, 1 mL supernatant was transferred to a new tube and 0.25 mL 1% TBA in 0.05 N NaOH was added. The tubes were then mixed and kept in a boiling water bath for 1 h. The concentration of the MDA-TBA complex was assessed by HPLC after its separation with ion exclusion and a reverse-phase C-18 column (5.0 mm × 250 mm, Waters, Milford, MA, USA). The UV/vis detector set at 532 nm. All data were collected and analyzed by an Empower Workstation 2.0 (Waters, Milford, MA, USA).

### GSH and GSSG detected by liquid chromatography/mass spectrometric assay (LC/MS)

GSH and GSSG contents were determined by liquid chromatography/mass spectrometric assay (LC/MS) based on a previous report [16]. After incubation, the blood samples were immediately centrifuged at 600 × g for 5 min (5804R, Eppendorf AG, Hamburg, Germany). The packed erythrocytes were then washed twice with equal volumes of ice-cold isotonic saline solution. The packed erythrocytes samples (400 μL) were added in ice-cold KCl solution (1.15%, 400 μL). The suspensions were lysed by freezing and thawing three times to ensure complete lysis. About 50 μL of the cell lysate was transferred to a 1.5 mL snap-cap conical-bottom centrifuge vial. The glutathione ethyl ester (20 μL, 0.01 mg/mL) was added to cell lysate that severed as an internal standard. The 2-Nitrobenzoic acid (100 μL, 10 mM; Sigma, St. Louis, MO, USA) was also added to cell lysate. MS analysis employed positive ion electrospray ionization (Agilent 6404, Santa Clara, CA, USA). The HPLC eluate was introduced into the stainless-steel electrospray capillary spray held at 2.3 kV. The source and detector voltages were 20 and 655 V. The low- and high-mass resolutions were set at 12.5 during analysis. Selected ion monitoring (SIM) was set to simultaneously monitor the ions with m/z of 505 and 613 (protonated molecular ions of GSH and GSSG). The HPLC conditions employed a Phenomenex C-18 column (5.0 mm × 500 mm, 411 Madrid Avenue, Torrance, CA, USA), mobile phase A (aqueous solution with 0.1% (v/v) trifluoroacetic acid) and mobile phase B (acetonitrile). At time zero, mobile phase A was pumped isocratically for 2 min. Mobile phase B was increased from 0% to 60% (from 2 min to 12 min) and held at 60% for an additional 2 min (all flow rates: 0.6 mL/min). The injection volume was 20 μL. All data were expressed as nmol/g hemoglobin (Hb) [16].

### The effects on membrane of human erythrocytes analyzed by flow cytometry

The effects of flaxseed oil on erythrocyte membrane were assessed as described in a previous report with some modifications [17]. After incubation, the cell suspension (4 × 10^5^) was washed twice with PBS. Then, the samples were suspended by binding buffer (300 μL) in tube and 5 μL Annexin V-FITC (Keygene, Nanjing, China) was added to cell suspension for cell labeling. The samples were examined by FACSCalibur flow cytometer (Becton-Dickinson, San Jose, CA, USA). The CellQUEST software programs (Becton-Dickinson, San Jose, CA, USA) were used for data analysis. Forward and sideward scatter profiles were used to define the region of the intact red cell population. The percentage of Annexin V-FITC positive erythrocytes was determined from the fluorescence signal in excess of that obtained with respect to a negative (unlabeled) control sample aliquot.

### Deformity of morphology of human erythrocytes assayed by ultra scanning electron microscopy (USEM)

The sample slides were prepared according to a previously method [18]. Three drops of blood cell suspension (5 × 10^6^) were directly dripped into phosphate buffer (0.05 M, pH 7.2, containing 2% glutaraldehyde solution). The test tube was gently inverted three times, and the cell suspensions were fixed for 1 h. The erythrocytes were settled by gravity. One drop (1 × 10^6^ cells) from the erythrocyte layer was dripped onto a piece of glass (2 cm × 2 cm). Erythrocytes adhered onto the sections that were dehydrated with 50%, 70%, 90%, and 100% ethanol until dry. After dehydration, the slides were coated with gold vapour (Eiko IB-3 Ion Coater, Tokyo, Japan). All sections of photos were magnified from 5000 to 25000 by ultra scanning electron microscopy (USEM, EDAX FEI QUANTA 200, Amsterdam, Holland).

### Fatty acid compositions of human erythrocytes membrane assayed by gas chromatography assay (GC)

Fatty acid compositions of erythrocytes membrane were detected by gas chromatography assay (GC) [19]. After incubation, the blood samples were washed three times by a 1-to-10 dilution with NaCl (0.15 M) before gas chromatography assay. The NaCl mixtures were centrifuged at 1000 × g for 10 min (4°C) and discarded supernatant (5804R, Eppendorf AG, Hamburg, Germany). The cell sample (200 μL) was added to distilled water (800 μL) and centrifuged at 956 × g for 10 min. The cell pellet, which contained the phospholipid membranes, was washed with distilled water (800 μL) and centrifuged again at 956 × g for 10 min. After the centrifugation, the supernatant was discarded and the cell pellet was suspended by distilled water (400 μL) and chloroform/methanol (3 mL, v/v, 1:1). The mixture was shaken for 10 min. Chloroform layer was transferred into another tube and solvent was removed by nitrogen evaporation. The phospholipids were simultaneously hydrolyzed and methylated with another mixture (toluene, 100 μL and BF3/MeOH, 500 μL) at heating block (100°C) for 60 min. After cooling, distilled water (800 μL) and hexane (800 μL) were added to mixture. The mixture was shaken for 5 min and mixture was settled on ice for 30 min. Hexane layer (upper layer) contained the methylated fatty acids that was transferred to gas chromatography vials and stored at −20°C until analysis. The samples were separated on a silica capillary column (30 m × 0.25 mm, 0.25 μm, Durabond chemically bond, DB-WAX, Agilent, CA, U.S.A) with a 6890 N network gas chromatograph and a 7683B series injector (Agilent, CA, USA). The Chemstaion Revision A.08.03 (Agilent, CA, U.S.A) was used for quantification and identification of peaks. The analytic conditions were used as follows: sample was injected 2 μL, carrier gas was 1.2 mL/min (nitrogen), injector temperature was set on 260°C, flame ionization detection was set on 275°C, split ratio was 1:20, oven temperatures were from 185°C to 245°C with a stepped temperature program and run times were set on 57–60 min.

### Statistical analysis

All data were presented as mean ± S.D. One-way ANOVA followed by the least significant difference point test was used to identify significantly different groups. *P* < 0.05 indicates significant differences among groups.

## Results

### Flaxseed oil decreased MDA concentration in human erythrocytes at high glucose level

**Figure 1:**
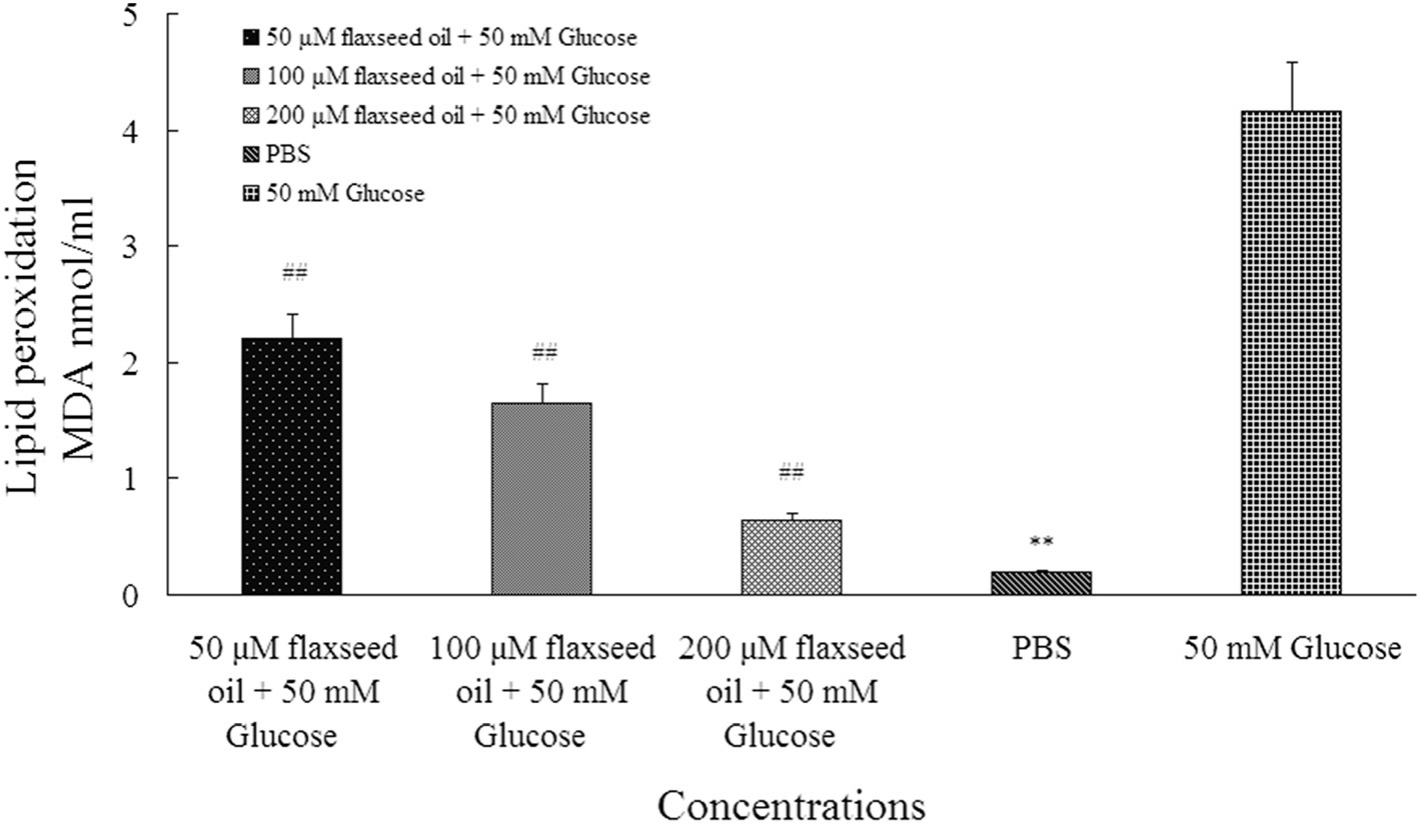
**demonstrated flaxseed oil reduced MDA level in erythrocytes.** The MDA level in three concentrations of flaxseed oil and PBS groups were remarkably lower than 50 mM glucose group. The ^##^ and ** indicate significant difference from 50 mM glucose group (n = 24, *P* < 0.01).

As displayed in Figure 1, the MDA level in PBS group was about 20-fold significantly lower than positive control group (0.2 ± 0.01 MDA nmol/mL versus 4.16 ± 0.62 MDA nmol/mL, R^*2*^ = 0.997, *P* < 0.01). Meantime, three doses of flaxseed oil obviously inhibited MDA levels that were about 1-fold, 1.5-fold and 2-fold lower than 50 mM glucose group respectively (R^*2*^ = 0.997, *P* < 0.01).

### Flaxseed oil maintained GSH and reduced GSSG in human erythrocytes at high glucose level

**Figure 2:**
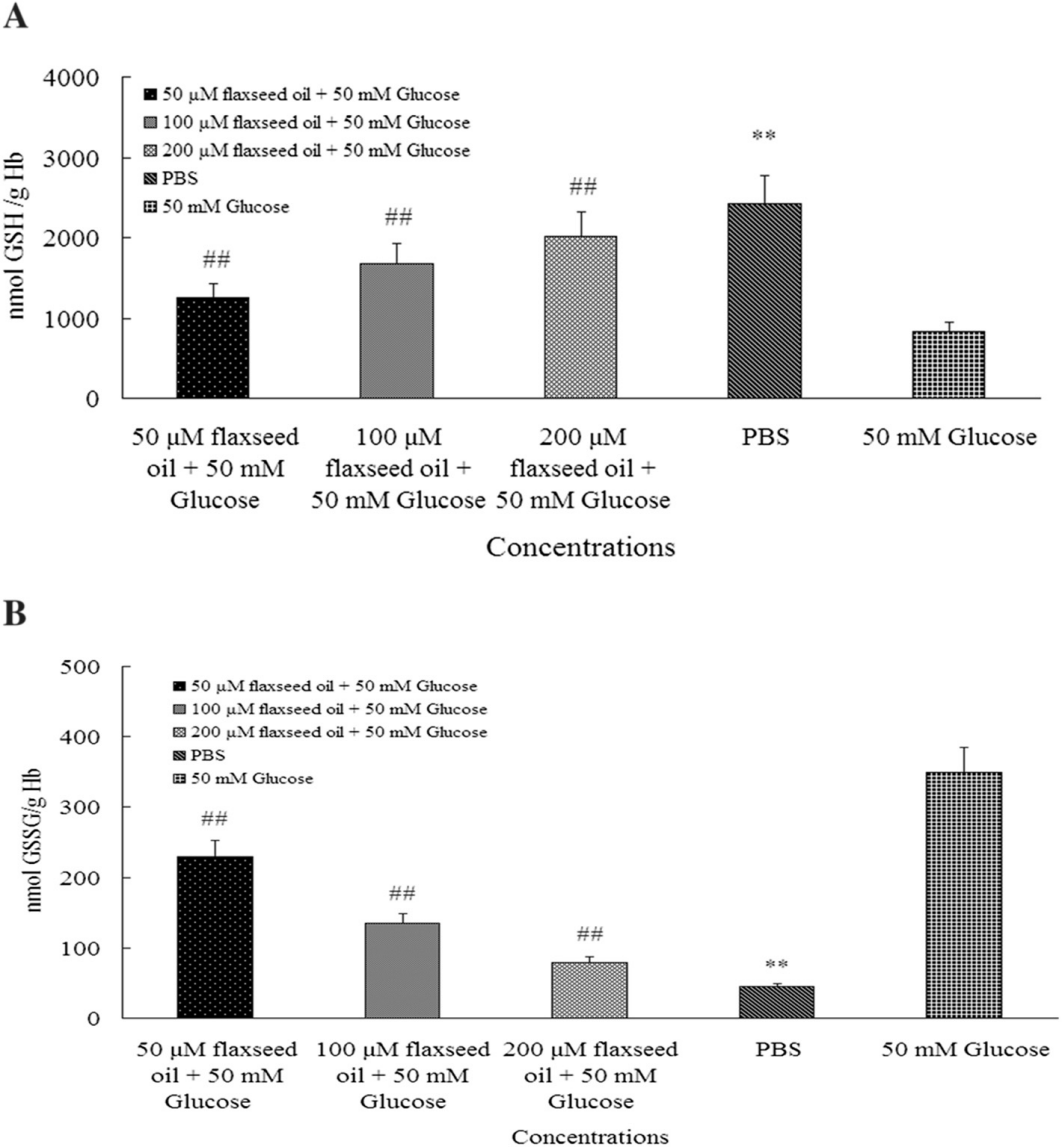
**A and B illuminated GSH and GSSG levels in erythrocytes.** The Figure 2 A demonstrated that flaxseed oil maintained GSH levels in erythrocytes. The GSH levels in three doses of flaxseed oil groups were obviously higher than 50 mM glucose group. The GSH level in PBS group was also higher than 50 mM glucose group. In the Fig.2 B, flax seed oil remarkably reduced GSSG level in flaxseed oil groups, and PBS group was also lower than 50 mM glucose group. The ^##^ and ** indicate significant difference from 50 mM glucose group (n = 24, *P* < 0.01).

As illustrated in Figure 2, the GSH level in PBS group was remarkably about 2.9-fold higher than positive control group (2416.25 ± 147.35 GSH nmol/g Hb versus 830.82 ± 96.76 GSH nmol/g Hb, R^2^ = 0.9985, *P* < 0.01). The GSH levels in three flaxseed oil groups were about 1-fold, 2-fold and 2.5-fold higher than 50 mM glucose group respectively. Furthermore, as demonstrated in Figure 2 B, three doses of flaxseed oil significantly inhibited GSSG levels that were about 1-fold, 2-fold and 3-fold lower than positive control group respectively (R^2^ = 0.9979, *P* < 0.01). The GSSG level in PBS group was also 8.3-fold lower than positive control group (21.67 ± 3.14 GSSG nmol/g Hb versus 181.74 ± 9.77 GSSG nmol/g Hb, R^2^ = 0.9979, *P* < 0.01).

### Flaxseed oil maintained phospholipids symmetry of membrane in human lymphocytes at high glucose level

**Figure 3:**
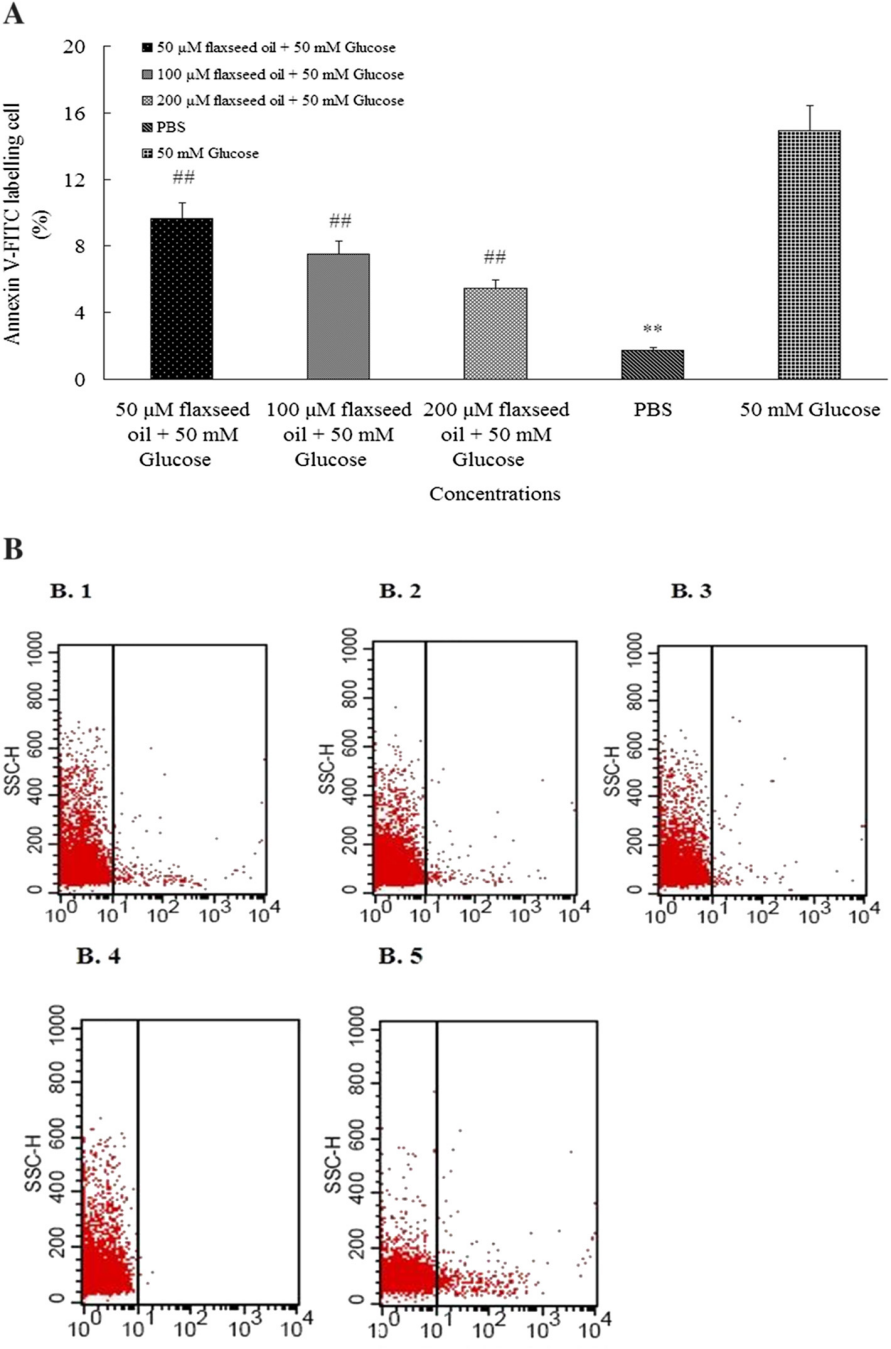
**A and B illustrated erythrocytes were labeled by FITC probe.** The Figure 3 A demonstrated that flaxseed oil protected membrane of erythrocytes. The three concentrations of flaxseed oil groups and PBS group were obviously labeled by FITC less than 50 mM glucose group (n = 24). The ^##^ and ** indicate significant difference from 50 mM glucose group (*P* < 0.01). The Figure 3 B 1–5 were representative figures of flow cytometry. The Figure 3 B 1–3 illustrated three concentrations of flaxseed oil protected on membrane. The PBS group (Figure 3 B 4) was also significant difference from 50 mM glucose group (Figure 3 B 5).

The fluorescent labeling level in PBS group was about 12-fold lower than 50 mM glucose group (1.13 ± 0.17% versus 13.95 ± 1.66%, *P* < 0.01) (Figure 3 A). The fluorescent labeling levels in three flaxseed oil groups were about 0.5-fold, 1-fold and 2-fold lower than 50 mM glucose group respectively (*P* < 0.01). Besides, as demonstrated in Figure 3 B1-B5 (representative images of flow cytometer), the fluorescent labeling levels in three flaxseed oil groups (Figure 3 B1-B3) and PBS group (Figure 3 B4) were obviously lower than 50 mM glucose group (Figure 3 B5).

### Flaxseed oil maintained normal cellular erythrocytes profile in human erythrocytes at high glucose level

**Figure 4:**
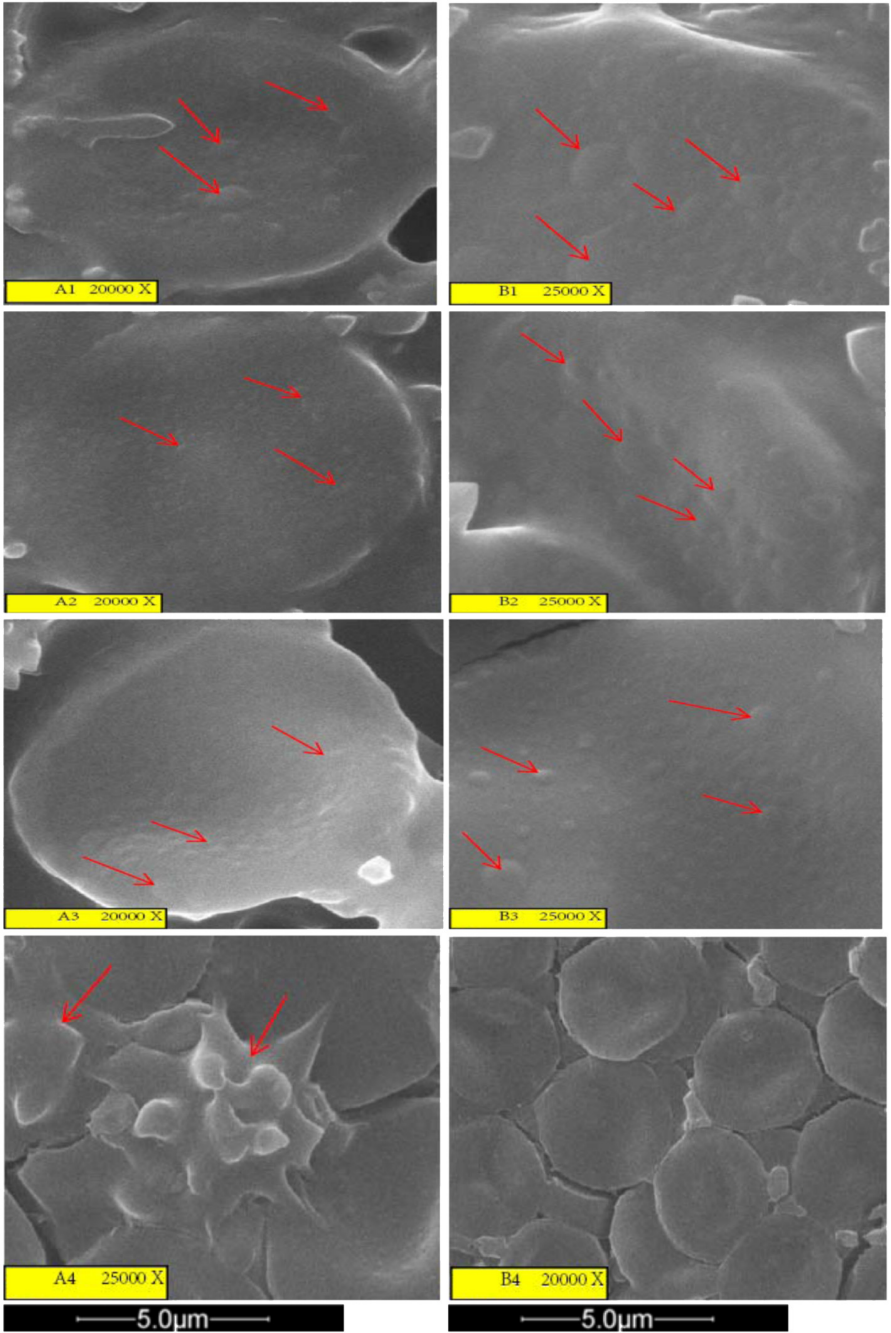
**Representative erythrocyte images under scanning electron microscopy.** In the Figure 4 A1 and B1, two figures demonstrated that the lowest dose of flaxseed oil exerted minor ability on membrane. Many bulges gradually emerged on membrane (red arrows, 20000× and 25000×). In the Figure 4 A2 and B2, 100 μM flaxseed oil group was better than 50 μM flaxseed oil group, the membrane gradually became to smooth and bulges also gradually alleviated on membrane (red arrows, 20000× and 25000×). In the Figure 4 A3 and B3 (20000× and 25000×), the 200 μM flaxseed oil group was similar as PBS group (Figure 4 B4, 20000×), the normal shape and normal membrane in PBS group. In the Figure 4 A4, the erythrocytes in positive group (50 mM glucose without flaxseed oil). The blood cells became flat cells (leptocyte) and acanthoid cells (25000×). They basically lost their normal shape.

As illustrated in Figure 4 (representative images of USEM). The Figure 4 A1 and B1 demonstrated that the lowest dose of flaxseed oil (50 μM) exerted minor effects on blood cells at high glucose level. Many bulges emerged on membrane (red arrows). The 100 μM group (Figure 4 A2 and B2) and 200 μM group (Figure 4 A3 and B3) obviously inhibited membrane damage and deformation that both were better than 50 μM flaxseed oil group and positive control group. The Figure 4 A4 illustrated that erythrocytes became to acanthocytes at the high glucose level. However, compared with positive control group, the human blood cells maintained normal shape in PBS group (Figure 4 B4).

### Flaxseed oil affected fatty acid composition of membranes in human erythrocytes at high glucose level

Fatty acid compositions of membranes were summarized in Table 1. In our results, the percentage of RBC C18:0 in negative control group was obviously lower than 50 mM glucose group (25.5 ± 1.7% versus 44.3 ± 3.9%, *P* < 0.01). The percentages of RBC C16:0, C18:0 and C18:1 in the 50 μM flaxseed oil group were also lower than positive control group respectively (*P* < 0.05). The percentages of RBC C22:0, C22:5 and C22:6 were higher than 50 mM glucose group (*P* < 0.05). Moreover, the percentages of RBC C16:0, C18:0 and C18:1 in 100 μM or 200 μM flaxseed oil groups were also remarkably lower than 50 mM glucose group (*P* < 0.01). The percentages of RBC C22:0, C22:5 and C22:6 also were significantly higher than positive control group (*P* < 0.01). Meanwhile, total concentration of membrane fatty acid in 50 μM flaxseed oil group was higher than 50 mM glucose group (18.29 ± 1.4 versus 17.04 ± 2.5 pmol/mg Hb, *P* < 0.05). And total concentration of membrane fatty acid in 100 μM and 200 μM flaxseed oil groups were also obviously higher than 50 mM glucose group (20.10 ± 2.2 and 27.89 ± 2.7 versus 17.04 ± 2.5 pmol/mg Hb, *P* < 0.01).

**Table 1:** The membrane fatty acid composition of erythrocytes (% of total FA)

	**50 μM flaxseed oil + 50 mM glucose**	**100 μM flaxseed oil + 50 mM glucose**	**200 μM flaxseed oil +50 mM glucose**	**glucose group (50 mM)**	**PBS(0.01 M)**
C14:0	2.90 ± 0.3*	3.70 ± 0.5**	4.20 ± 0.7**	1.90 ± 0.2	6.80 ± 0.4^##^
(Myristic acid)					
C16:0 (Palmitic acid)	36.44 ± 1.8*	27.25 ± 2.9**	24.22 ± 1.9**	38.10 ± 2.7	19.70 ± 2.2^##^
C17:0	0.61 ± 0.1	0.62 ± 0.2	0.67 ± 0.1	0.62 ± 0.1	0.64 ± 0.2
(Internal standard)					
C18:0	19.16 ± 3.0*	14.81 ± 3.0**	10. 44 ± 3.3**	21.30 ± 3.9	5.50 ± 1.7^##^
(Stearic acid)					
C18:1	21.11 ± 2.2*	19.01 ± 4.1**	16.71 ± 2.9**	24.40 ± 2.4	14.40 ± 0.9^##^
(Vaccenic acid)					
C18:2	2.20 ± 0.3*	4.90 ± 0.6**	6.30 ± 0.8**	1.40 ± 0.2	9.60 ± 0.5^##^
(Linoleic acid)					
C22:0	0.97 ± 0.2*	1.29 ± 0.2**	2.00 ± 0.4**	0.90 ± 0.1	3.90 ± 0.3^##^
(Behenic acid)					
C22:5	0.77 ± 0.2*	1.40 ± 0.3**	1.90 ± 0.3**	0.70 ± 0.1	2.80 ± 0.2^##^
(Docosapentaenoic acid)					
C22:6	0.79 ± 0.1*	1.10 ± 0.1**	2.10 ± 0.1**	0.60 ± 0.1	3.20 ± 0.6^##^
(Docosadienoic acid)					
RBC fatty acids	18.29 ± 1.4*	20.10 ± 2.2**	27.89 ± 2.7**	17.04 ± 2.5	32.33 ± 3.1^##^
total concentration					
(pmol/mg Hb)					

**Note**: Three concentrations of flax seed oil effect on fatty acid composition of membrane. ^*^: *P* < 0.05, ^**^ and ^##^: *P* < 0.01 are significantly different from 50 mM glucose group (n = 24).

## Discussion

Hyperglycemia probably induce oxidative stress because of excessive oxygen radical production caused by the auto-oxidation of glucose or stimulation of cytochrome P450-like activity, which results from excessive NADPH production by glucose metabolism [6]. This study indicated that flaxseed oil could reduce the lipid peroxidation level (MDA) and GSSG, and maintain the GSH contents in erythrocytes. In addition, Flaxseed oil could also maintain the normal cellular shape in human erythrocytes exposed to high glucose model.

MDA is an oxidative modification of cellular macromolecules, which can induce cell apoptosis, cell necrosis, as well as tissue damage [18]. MDA molecules may cross-link with membrane proteins or membrane phospholipids to form polymers. Thereby, these polymers can induce abnormal morphology and malfunction of cells [18-20]. In the present study, flaxseed oil reduced MDA level in human erythrocytes at high glucose level. This phenomenon indicated that flaxseed oil contained a high content of n-3 PUFA and many double bonds in n-3 PUFA avoided damage on membrane form oxidative stress [18]. Membrane incorporation of PUFAs may reduce cellular susceptibility to lipid peroxidation [19], alter membrane fluidity [19], enhance receptor function, elevate enzyme activity [19,21] and influence the production of lipid mediators [19,21]. The observation was probably related to the reaction of free radicals with methylene groups in PUFA to avoid free radical conjugating with fatty acids of membrane [7,22,23].

GSH is the most abundant antioxidant in human body [23,24]. As erythrocytes contain more than 95% of the blood GSH [23,24], GSH plays a critical role for protecting erythrocytes against oxidative stress [23-26]. A previous study observed that flaxseed oil could transfer phenolic hydrogen to a peroxyl free radical of a peroxidized PUFA [23]. This mechanism can inhibit the radical chain reaction for preventing the peroxidation of PUFA in cellular or subcellular membrane phospholipids [23]. Meanwhile, several studies in diabetic models also showed that glucose could penetrate intracellularly to activate polyol pathway and evoke depletion of NADPH [23-29]. In this study, our results showed that flaxseed oil reduced GSSG level and maintained GSH level in erythrocytes, suggesting it may enhance antioxidant capacity in human erythrocytes. Similar findings were reported in a previous study in erythrocytes of diabetic rats [30].

Phospholipid symmetry and membrane integrity are key factors in the survival of cells during oxygen deprivation [7,8]. Under hypoxia condition, change in the membrane integrity is a symptom of injury. It can be measured as changes in the lipid content and composition [8,11,]. In many clinical researches, membrane perturbation can be deemed as a result of oxidative stress [8,31-33]. were related to phosphatidylserine (PS) externalization on membrane symmetry caused by excess free radicals [33]. High glucose exposure distorted erythrocytes to form acanthocytes [8, 32 ,33, 34]. Thus, our results indicated that flaxseed oil could counteract lipid peroxidation to enhance fluidity or prevent phosphatidylserine externalization on membrane [32]. Meantime, images of ultra scanning electron microscopy showed that flaxseed oil could maintain cellular shape of erythrocytes at high glucose level and results of flow cytometry also illustrated similar results. These results suggested that flaxseed oil could maintain normal phospholipids symmetry on membrane of erythrocytes [8,32-34].

The membrane of the red blood cell plays many roles that aid in regulating their surface deformability, flexibility, adhesion to other cells and immune recognition [35-37]. These functions are highly dependent on its composition, which defines its properties [35]. Half of the membrane mass in human and most mammalian erythrocytes are proteins. The other half is lipids, namely phospholipids and cholesterol [36]. The erythrocyte cell membrane comprises a typical lipid bilayer, similar to what can be found in virtually all human cells [37]. This lipid bilayer is composed of cholesterol and phospholipids in equal proportions by weight. The lipid composition is important as it defines many physical properties such as membrane permeability and fluidity [37]. Unlike cholesterol which is evenly distributed between the inner and outer leaflets, the 5 major phospholipids are asymmetrically disposed (outer monolayer: phosphatidylcholine, PC and sphingomyelin, SM; inner monolayer: phosphatidylethanolamine, PE, phosphoinositol, PI and phosphatidylserine, PS) [17,37,38]. In the present study, concentrations of PUFA C18:2, C22:5 and C22:6 were decreased in membrane while concentrations of monounsaturated fatty acid (MUFA) C14:0 and C18:1 were dramatically increased, which resulted in a net higher concentration of total monounsaturated fatty acids in the positive control group [19,39]. Moreover, earlier studies also found that the carbonyls of C18:1 and C16:0 were exposed to the aqueous interface of phospholipids, the C18:1 and C16:0 on membranes may result in increase of rigid molecular order on membrane structures [19,39-42]. The worse partition of the fatty acid compositions in the lipid bilayer may alter the biochemical properties of the cell membrane, the membrane shape, organization, and permeability [10,39,41-46]. Therefore, our results indicated that flaxseed oil could protect against to the deformability of human erythrocytes.

## Conclusion

Our study found that flaxseed oil could attenuate lipid peroxidation, preserve anti-oxidation capacity and maintain fatty acid compositions of in membrane of human erythrocytes. The flaxseed oil could also inhibit phosphatidylserine externalization of membrane. More studies on detailed molecular mechanisms are warranted.

## Abbreviations

(PUFA), Polyunsaturated fatty acid; (ALA), α-linolenic acid; (MeOH), Borontrifluoride (BF_3_) / methanol; (DHA), Docosahexaenoic acid; (EPA), Eicosapentaenoic acid; (EDTA), Ethylenediamine tetraacetic acid; (GC), Gas chromatography assay; (Hb), Hemoglobin (HPLC), High performance liquid chromatography; (LDL), Low-density lipoprotein; (GSH), L-glutathione reduced; (GSSG), L-glutathione oxidized; (LC/ MS), Liquid chromatography/mass spectrometric assay; (MDA), Malondialdehyde; (PC), Phosphatidylcholine; (PE), Phosphatidylethanolamine; (PI), Phosphoinositol; (PS), Phosphatidylserine; (SM), Sphingomyelin; (TBA), Thiobarbituric-acid; (USEM), Ultra scanning electron microscopy.

## Competing interest

The authors declare that there is no conflict of interest.

## Authors’ contributions

WY designed the experiments, analyzed the data and drafted the manuscript. WY, JF, DW and MY operated all experiments. Qd H, Jq X and Qc D prepared oil samples. All authors read and approve the final manuscript.

## Supplementary Material

Additional file **Table S1.** Fatty acid compositions of flax seed oil. Click here for file
