# Supplementary material for: Effects of flaxseed oil on anti-oxidative system and membrane deformation of human peripheral blood erythrocytes in high glucose level
Source: Lipids Health Dis. 2012 Jul 8;11:88. doi: 10.1186/1476-511X-11-88 (PMC3459705; doi:10.1186/1476-511X-11-88)
Supplement: Additional file — Table S1. Fatty acid compositions of flax seed oil. [file 1476-511X-11-88-S1.doc]

Table. 1 Fatty acid compositions of flax seed oil

| Fatty acid compositions （%） |
| --- |
| Palmitic acid 5.66  Stearic acid 4.21  Oleic acid 20.14  Lionleic acid 14.54  α-lionleic acid 55.46 |
